# Hybrid Loss-Constrained Lightweight Convolutional Neural Networks for Cervical Cell Classification

**DOI:** 10.3390/s22093272

**Published:** 2022-04-24

**Authors:** Wen Chen, Weiming Shen, Liang Gao, Xinyu Li

**Affiliations:** State Key Laboratory of Digital Manufacturing Equipment & Technology, Huazhong University of Science and Technology, Wuhan 430074, China; chenwen0704@hust.edu.cn (W.C.); gaoliang@mail.hust.edu.cn (L.G.); lixinyu@mail.hust.edu.cn (X.L.)

**Keywords:** medical imaging, deep learning, lightweight convolutional neural networks, cervical cancer diagnosis, hybrid loss function

## Abstract

Artificial intelligence (AI) technologies have resulted in remarkable achievements and conferred massive benefits to computer-aided systems in medical imaging. However, the worldwide usage of AI-based automation-assisted cervical cancer screening systems is hindered by computational cost and resource limitations. Thus, a highly economical and efficient model with enhanced classification ability is much more desirable. This paper proposes a hybrid loss function with label smoothing to improve the distinguishing power of lightweight convolutional neural networks (CNNs) for cervical cell classification. The results strengthen our confidence in hybrid loss-constrained lightweight CNNs, which can achieve satisfactory accuracy with much lower computational cost for the SIPakMeD dataset. In particular, ShufflenetV2 obtained a comparable classification result (96.18% in accuracy, 96.30% in precision, 96.23% in recall, and 99.08% in specificity) with only one-seventh of the memory usage, one-sixth of the number of parameters, and one-fiftieth of total flops compared with Densenet-121 (96.79% in accuracy). GhostNet achieved an improved classification result (96.39% accuracy, 96.42% precision, 96.39% recall, and 99.09% specificity) with one-half of the memory usage, one-quarter of the number of parameters, and one-fiftieth of total flops compared with Densenet-121 (96.79% in accuracy). The proposed lightweight CNNs are likely to lead to an easily-applicable and cost-efficient automation-assisted system for cervical cancer diagnosis and prevention.

## 1. Introduction

Cervical cancer is the fourth most frequently diagnosed cancer and the fourth leading cause of cancer death worldwide in women; in 2020, more than 600,000 women were diagnosed and more than 340,000 deaths were caused by cervical cancer [1]. In particular, cervical cancer is the most commonly diagnosed cancer and the leading cause of cancer death in many developing countries. The human development index (HDI) and poverty rates have been shown to account for >52% of global variance in mortality [2]. Cervical cancer is the rare end stage of an unresolved human papilloma virus (HPV) infection. The time lag between the peak of HPV infection and the peak of cancer incidence is two to four decades, so the ability to detect cancer in the early phase will enable early effective intervention or prevention. The Pap smear has been extensively adopted in developed countries and is credited with reducing the mortality rate of cervical cancer significantly. However, only 44% of women in lower-middle income countries (LMICs) have ever been screened for cervical cancer, compared with >60% in high-income countries [3]. Because of the lack of health resources and the scarcity of qualified medical technicians, women in these countries are the true high-risk group for cervical cancer.

To address these issues, automation-assisted screening systems have been researched and applied to enhance efficiency and increase the availability of cervical cancer screening. Over the last ten years, automated image analysis techniques have been adopted for building automation-assisted cervical screening systems. The systems generally comprise three steps: cell segmentation, feature extraction or feature selection, and cell classification. Several methods aimed at addressing cell segmentation have been proposed and employed, such as fuzzy C-means (FCM) clustering [4], adaptive threshold decision method [5], iterative threshold methods [6], morphological operation, and watershed transformation [7]. After the segmentation step, important features are extracted from cell components. Several researchers have investigated this feature extraction topic. RF (random forest) algorithm [8], a new elongated quinary pattern (EQP) method [9], and a three-layered learning model [10] were proposed for the purpose of finding a reliable set of features. Furthermore, an algorithm that combines nearest neighbor techniques and a GA was proposed for feature selection [11]. Subsequently, different features were calculated and obtained, for example: features derived from 2-D Fourier transform spectrum and log transforms of images [8]; features based on various biologically interpretable, clinically significant shapes, as well as morphology [12]; features including shape, statistical Gabor and Markov random fields features [13]; features based on the texture and structure of cytoplasm [14]; and features based on texture and shape [15]. These selected features were then adopted for classifying cervical cell images. Additionally, a classification method that combines global significance value, texture statistical features, and time–series features was proposed for cervical cell classification [16].

Inspired by the great success of deep learning technology in various computer vision and pattern recognition tasks [17,18,19], the application of deep convolution neural networks in the field of biomedicine has increased [20,21,22]. Convolutional neural networks (CNNs) benefit automation-assisted cervical cancer screening systems in many ways [23,24]. In the research, we briefly introduce those deep learning-based approaches. DeepPap [25] was proposed to extract deep features automatically for classifying cervical cells. The morphology information was added in another CNN-based approach [26]. A hybrid transfer learning algorithm that combines features from different CNNs was presented in [27]. Furthermore, a hierarchical modular neural network architecture [28] for automated screening of the cervical cancer was also explored. A comparative study was conducted on five CNNs [29] to check their classification performances. Graph convolutional network (GCN) features were combined with CNN features [30] to improve the classification performances. The fused deep CNN features that were extracted from several different models are combined with AF-SENet for cervical tissue pathological images classification [31]. Mask-RCNN was utilized for collected segmented image patches, and then these image patches were classified by a VGG-like model [32]. An improved YOLOv3 was proposed with soft-scale anchor matching [33], which eliminates the traditional segmentation phase. Three deep CNNs were trained respectively, and then ensemble features together for prediction [34]. A transfer learning method based on knowledge distillation was proposed and evaluated using the Herlev dataset [35].

Among these approaches, a number of CNN architectures [36,37,38] have been tested and applied to the task of cervical cell classification. However, many former approaches targeting the improvement of classification accuracy do not consider the computational costs, which can be a big hindrance for the worldwide application of automation-assisted screening systems. CNNs often have a large number of parameters and require massive floating-point operations to achieve satisfactory accuracy. Therefore, deep and powerful CNNs require significant memories and hardware resources. Furthermore, fine-grained cervical cell classification has superior clinical significance that can ultimately facilitate subsequent evaluation and follow-up treatment. However, most of the previous studies only change the number of classes in CNNs for the fine-grained classification without further modifications. The key to fine-grained classification lies in improving the discriminative power of CNN models, especially when it comes to confusable samples. Indistinguishable samples from different classes should be considered when designing automation-assisted screening systems.

With the proposed method, two lightweight CNN architectures can perform at the same level as typical, large-sized CNNs. Furthermore, they achieve the same results at a much lower computational cost. In this paper, several well-designed lightweight CNNs are adopted to reduce hardware requirements and computational costs. A more efficient method inspired by triplet loss [39] is also proposed for solving the problem of confusing samples. Unlike the traditional loss function, which computes the discrepancy between the prediction and the true targets, the proposed method utilizes a hybrid loss function, adding functions that making the features from the same class closer while making features from different classes farther away from each other. To the best of our knowledge, this is the first use of triplet loss as well as lightweight Ghostnet model for this fine-grained cervical cell classification task.

This paper proposed a hybrid loss function with label smoothing to improve the distinguishing power of lightweight convolutional neural networks applied in cervical cell classification. We believe that this work has following contributions:

1. The proposed hybrid loss function allows the lightweight CNNs to obtain enhanced classification results by improving the ability of CNNs to distinguish confusing samples among the cervical cell images.
2. The integration of the proposed hybrid loss function with lightweight CNN models provides some significant value in practical applications under limited computational resources.

The remainder of this paper is organized as follows: Section 2 describes the proposed method for cervical cell classification under limited resources. Section 3 presents the experiment results and analyses. Section 4 summarizes the conclusions and discusses the directions for future work.

## 2. Proposed Method

This paper proposes a hybrid loss function for cervical cell classification in a computer-aided screening system, which aimed to improve the distinguishing power of the CNN models, especially when encountering confusing samples. Several lightweight CNNs were selected and trained with the hybrid loss function to choose economic and effective models.

### 2.1. Overview

For this fine-grained problem, the goal of this study is to improve the distinguishing power of the automation-assisted system under restrained computation resources, which will achieve economic feasibility as well as technical reliability for worldwide application in cervical cancer screening systems.

For the purpose of improving the distinguishing power of the system, the triplet loss is utilized as a component of the proposed hybrid loss function. Under the condition of joint supervision, on one hand, the differences in inter-class features are enlarged. On the other hand, the variations in intra-class features are reduced.

Four lightweight CNN architectures are selected and investigated to address the constraint of limited resources. These lightweight models have great capability for building competent systems with small computation requirements.

**Figure 1:**
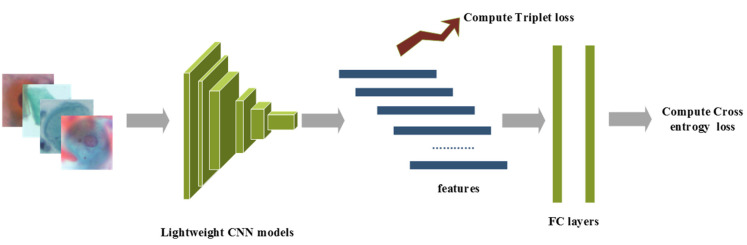
The overview of the proposed method.

The overview of the proposed method is presented as follows: raw images after pre-processing are sent into the lightweight CNN model, which is initialized with pre-trained ImageNet weights. The CNN features after the last convolutional layer are adopted for calculating triplet loss, while class prediction logits are adopted for computing cross-entropy loss, and a hyperparameter is used to balance these supervision signals. Furthermore, label smoothing is implemented to prevent overfitting. The details of the four lightweight CNN networks are discussed in Section 2.3. The overview of the proposed method is shown in Figure 1.

### 2.2. Hybrid Loss Function

The proposed hybrid loss function includes two losses as follows: cross-entropy loss and triplet loss.

(1) $$L=L_{Cross\;Entropy\;Loss}+\lambda L_{Triplet\;Loss}$$

*λ* is the balanced weight of triplet loss. The hybrid loss function aims to gradually make the average intra-class distance smaller and smaller, but make the average inter-class distance larger and larger. On one hand, the cross-entropy operation computes the cross-entropy loss between network predictions and target values. Intuitively, it forces the deep features of different classes to stay apart. On the other hand, in the learned feature space, the triplet loss function aims to pull the instances of same class closer, and at the same time pushing the instances belonging to different classes farther from each other.

The cross-entropy loss is computed as:

(2) $$L_{Cross\;entropy\;}=-\overset{N}{\underset{i=1}{\sum }}p\left(x_{i}\right)\log\left(q\left(x_{i}\right)\right)$$

where p is the true distribution of the image label, q is the distribution of image as predicted by the model, and N is the number of samples.

The triplet loss minimizes the distance between an image *x_i_^a^ (anchor)* and an image *x_i_^p^ (positive)*, both of which have the same class, and maximizes the distance between the *x_i_^a^(anchor)* and an image *x_i_^n^ (negative)* of a different class. The embedding is represented by $f\left(x\right)\in R^{d}$. Thus, the goal of the triplet loss is computed as:

(3) $$\|f\left(x_{i}^{a}\right)-f{(x_{i}^{p})\|}_{2}^{2}+\alpha <\|f\left(x_{i}^{a}\right)-f{(x_{i}^{n})\|}_{2}^{2}$$

$$\forall \left(f\left(x_{i}^{a}\right),f\left(x_{i}^{p}\right),f\left(x_{i}^{n}\right)\right)\in K$$

The triplet loss is computed as:

(4) $$L_{Triplet}=\overset{N}{\underset{i}{\sum }}{\left[\|f\left(x_{i}^{a}\right)-f{\left(x_{i}^{p})\right\|}_{2}^{2}-\|f\left(x_{i}^{a}\right)-f{(x_{i}^{n})\|}_{2}^{2}+\alpha \right]}_{+}$$

where $\|f\left(x_{i}^{a}\right)-f{(x_{i}^{p})\|}_{2}^{2}$ and $\|f\left(x_{i}^{a}\right)-f{(x_{i}^{n})\|}_{2}^{2}$ are feature distances of a positive pair and a negative pair, *α* is a margin between positive and negative pairs. *Κ* is the set of all possible triplets in the training set and has cardinality *N*; in this paper, *α* is set to 1.

### 2.3. Lightweight CNN Models

In this study, four lightweight CNN models (Squeezenet [40], MobilenetV2 [41], ShufflenetV2 [42], and Ghostnet [43]) are selected and evaluated to address the constraint of limited resources. These lightweight models have great capability of building competent systems with low computation requirements.

Squeezenet was proved to have comparable classification results with 50× fewer parameters than AlexNet. The distinctive trait of Squeezenet is the fire module, which follows three strategies: replace 3 × 3 filters with 1 × 1 filters; decrease the number of input channels to 3 × 3 filters; and downsample late in the network. These strategies enable the Squeezenet to decrease the quantity of parameters in the CNN model while maintaining a competitive accuracy.

MobilenetV2 allows a very memory-efficient inference and relies on the utilization of standard operations. The architecture benefits greatly from a novel layer module: the inverted residual with linear bottleneck. This module takes an input as a low-dimensional compressed representation which is first expanded to high dimension and filtered with a lightweight depth-wise convolution. Features are subsequently projected back to a low-dimensional representation with a linear convolution.

ShufflenetV2 inherited two operations from ShufflenetV1 [44]: pointwise group convolution and channel shuffle. These operations reduce computational cost while having very little impact on accuracy. ShuffleNetV2 further considers the actual speed on target hardware for compact model design. It introduces a channel split operator in the ShufflenetV2.

Ghostnet was proposed to build efficient neural architecture with high performance. The basic Ghost module splits the original convolutional layer into two parts and utilizes fewer filters to generate several intrinsic feature maps. Then, a certain number of cheap transformation operations can be further applied for generating ghost feature maps efficiently.

### 2.4. Training Strategy

The Pytorch deep learning framework is utilized for leveraging the implementation and experimentation of the proposed method. Stochastic gradient descent with a momentum of 0.9 is employed for optimizing the model. The weight decay is set to 0.005 without dampening. The initial learning rate is set to 0.0001 and is decreased by 0.1 at the 30th epoch and 60th epoch, respectively. The number of training epoch is 100, and the batch size is 16. Label smoothing [45] is adopted to prevent overfitting for a classification task.

## 3. Experiments and Analyses

In this section, the classification performances of the hybrid loss-constrained lightweight CNNs are investigated towards the fine-grained cervical cell classification task. Experiments on different loss constraints are conducted using several lightweight CNNs to illustrate the effectiveness of the proposed hybrid loss. Furthermore, comparisons between the proposed approach and several other CNN-based approaches are provided to illustrate the advantages of the effective lightweight CNNs in clinical applications.

### 3.1. Dataset and Pre-Processing

**Figure 2:**
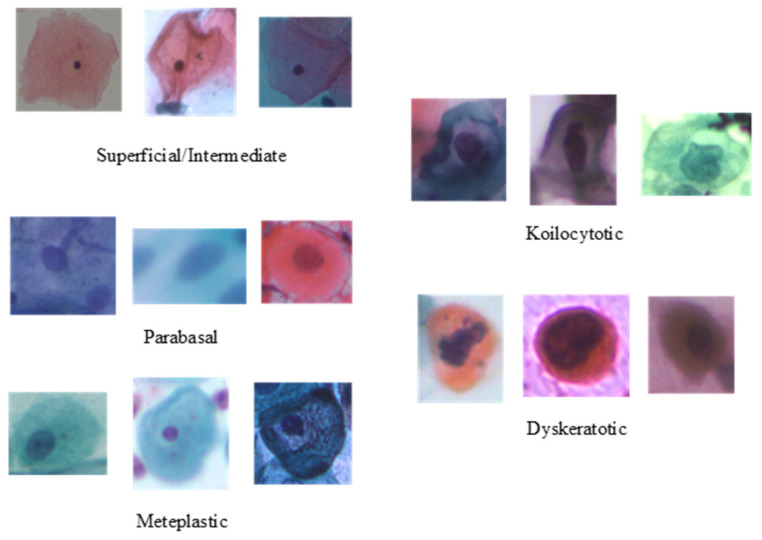
Samples categories from the SIPaKMeD dataset.

To evaluate the performance of the proposed method, the publicly available cervical cell image dataset SIPaKMeD [46] is adopted. It contains 4049 image samples of isolated cells, which have been cropped from 996 cluster cell images of Pap smear slides manually. The original sizes of these patches vary from each other. These images were acquired through a charge-coupled device (CCD) camera adapted to an optical microscope. The image samples are annotated by expert cytopathologists into five classes, depending on their cellular appearance and morphology: two classes for normal cells (superficial–intermediate and parabasal); two classes for abnormal cells (koilocytotic and dyskeratotic); and one class for benign cells (metaplastic). Sample images of individual classes are presented in Figure 2, and the class-wise distribution of the cell images is listed in Table 1.

The images (raw images with background) of SIPaKMeD are resized and center cropped into 224 × 224 pixels to facilitate the training phase of the CNN models. Rotate the image with the degree randomly selected from the range (−180, +180). Horizontal flip each image randomly with a given probability of 0.5. The image samples are reshuffled at every epoch.

ColorJitter is utilized for improving the diversity of the limited samples. It is an API function in Pytorch for image transformation, which aims to change the brightness, contrast, saturation, and hue of an image randomly. In our work, the value of the parameters in ColorJitter are set to 0.3, 0.5, 0.3, and 0.1, respectively:

- brightness (float or tuple of float (min, max)): How much to jitter brightness. The brightness_factor is chosen randomly from [max(0, 1 − brightness), 1 + brightness]
- contrast (float or tuple of float (min, max)): How much to jitter contrast. The contrast_factor is chosen randomly from [max(0, 1 − contrast), 1 + contrast]
- saturation (float or tuple of float (min, max)): How much to jitter saturation. The saturation_factor is chosen randomly from [max(0, 1 − saturation), 1 + saturation]
- hue (float or tuple of float (min, max)): How much to jitter hue. The hue_factor is chosen randomly from [−hue, hue]

Each image is then decoded into 32-bit floating point raw pixel values in [0, 1]. Then, the pixel intensity distribution of each input image is normalized by subtracting its mean and dividing the resulting difference by its standard deviation. A five-fold cross-validation method was adopted to report the classification performance for the SIPaKMeD dataset. Concretely, four-fifths of the image samples are used as the training set and the remaining samples as the validation set for five rounds. The classification evaluation metrics are obtained by averaging results from the five rounds.

### 3.2. Performances of Different Losses on Several Lightweight CNNs

To test the capability of the proposed method, two different losses are adopted and evaluated on four lightweight CNN models. The first is denoted by traditional loss: cross-entropy loss. The second is a hybrid loss for adding a triplet loss before the fully connected layers in the CNNs, denoted by hybrid loss: cross-entropy loss + triplet loss. The hyperparameter in the hybrid loss is set to 1 for all the selected lightweight CNNs. Four lightweight CNNs, Squeezenet, MobilenetV2, ShufflenetV2, and Ghostnet are trained under these two different constraints. Table 2 shows the classification performances (accuracy, precision, recall, and specificity) of all the models with traditional loss and hybrid loss, respectively.

Compared with lightweight CNNs trained under different constraints of losses, three lightweight CNNs (Squeezenet, ShufflenetV2, and Ghostnet) trained with proposed hybrid loss outperformed the models trained with traditional loss. As reported in Table 2, hybrid loss-constrained Ghostnet achieved the highest accuracy, precision, recall, and specificity among all the models. ShufflenetV2 acquired comparable classification performances. However, MobilenetV2 did not enhance results in the same way as other lightweight CNNs. These differences can be explained in part by the hyperparameter settings in the hybrid loss or the training of CNN models. From the comparison above, it can be regarded that utilizing the proposed hybrid loss function benefits the discriminative ability of several lightweight CNNs for cervical cell classification.

**Figure 3:**
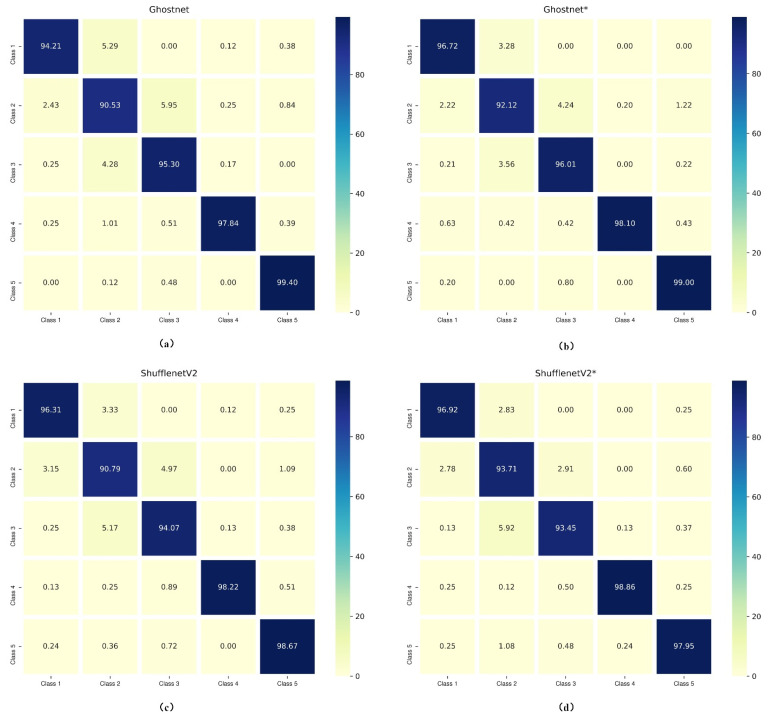
Confusion matrixes of Ghostnet and ShufflenetV2 trained with and without hybrid loss (“*” means the CNN model was trained with hybrid loss function). Class 1, superficial/intermediate; Class 2, parabasal; Class 3, metaplastic; Class 4, koilocytotic; Class 5, dyskeratotic. (**a**) Ghostnet; (**b**) Ghostnet*; (**c**) ShufflenetV2; (**d**) ShufflenetV2*.

Confusion matrices of the lightweight models (Ghostnet and ShufflenetV2) trained with different losses are presented in Figure 3. They display a detailed visualization of the classification performances on each cell class. As for the analysis of Ghostnet shown in Figure 3a,b, the normal cells (i.e., superficial–intermediate and parabasal), the benign cell (metaplastic), and the abnormal cells (koilocytotic) have better classification results in Ghostnet trained with hybrid loss function (Ghostnet*) than trained with traditional loss function (Ghostnet). As reported in Figure 3c,d, the classification results of ShufflenetV2 trained with hybrid loss function in terms of three classes (superficial–intermediate, parabasal, and koilocytotic) surpass the results of ShufflenetV2 trained with the traditional loss. Besides, the misclassification rate of parabasal in ShufflenetV2* is 2.92% lower than in ShufflenetV2. One explanation is that the proposed approach let the model learn more discriminative features. The hybrid loss enhances intra-class compactness and interclass separability in the Euclidean space. Therefore, the representative features generated by the hybrid loss can enhance the discriminative ability of some lightweight CNNs.

### 3.3. Comparisons with State-of-the-Art Methods

For the purpose of demonstrating the advantages of the proposed method, several other CNN based methods were selected for comparison. To analyze the requirements of computational cost for different models, the following metrics are calculated: (1) total parameters, (2) total memory, (3) total flops, with (4) accuracy is also listed as an important indicator. Total parameters reports the number of network parameters; total memory reports the memory usage of models; total flops reports floating point operations, which indicates the complexity of CNN models in inference; and accuracy reports the overall percentage of correctly identified cells.

Table 3 shows the comparison results of our proposed model with existing methods. All of these listed methods except the GCN method utilize only one single CNN model in an end-to-end manner. The GCN method combines the CNN features with GCN features. Overall, the results presented below show the lightweight ShufflenetV2 and Ghostnet trained with the hybrid loss function surpass the single Alexnet, VGG and Resnet-101models, and make a comparable result compared to the single DenseNet-121 model. However, from the aspect of computation requirements, these two lightweight models outperform all the other models by great superiority. As reported in Table 3, with the proposed method, ShufflenetV2 obtained a satisfactory classification result with only one-seventh of the memory usage, one-sixth of the number of parameters, and one-fiftieth of total flops compared with Densenet-121. Furthermore, the Ghostnet also reported comparative results.

From the above analyses, it can be concluded that the lightweight ShufflenetV2 and Ghostnet trained under the proposed hybrid loss can provide satisfactory classification performances with much lower computational cost. The experimental results fulfill the main goals of the proposed method, which is to enhance the discriminating power of the deeply learned features. These observations also provide compelling evidence that lightweight Ghostnet and ShufflenetV2 achieve satisfactory classification performances under limited resources. It can be stated that these experiments have proved the effectiveness and economic efficiency of the proposed methods.

## 4. Conclusions

This paper proposes the use of hybrid loss-constrained lightweight CNNs for fine-grained cervical cell classification. With the proposed joint supervision of hybrid loss function, the representation ability of CNNs for cervical cell classification is enhanced. This finding also confirms the usefulness of the lightweight CNN models with low computational cost. With the proposed method, ShufflenetV2 obtained satisfactory classification (96.18% accuracy, 96.30% precision, 96.23% recall, and 99.08% specificity) results with only one-seventh of the memory usage, one-sixth of the number of parameters, and one-fiftieth of the total flops compared with Densenet-121 (96.79% accuracy). GhostNet acquired an improved classification result (96.39% accuracy, 96.42% precision, 96.39% recall, and 99.09% specificity) with one-half of the memory usage, one-quarter of the number of parameters, and one-fiftieth of total flops compared with Densenet-121 (96.79% accuracy). It is believed that these results are an excellent initial step towards sample characterization in cervical cells images using deep learning under limited resources.

Nevertheless, the proposed method has a few notable limitations. First, the current study was not specifically designed for an end-to-end cervical cell screening system, and the detection of isolated cervical cells is another challenging task in this area. The lightweight CNN based system for cervical cells detection and classification is promising and significant for end-to-end automatic screening systems. Second, the collection of large amounts of labeled data is still a hindrance to the application of deep learning algorithms in healthcare areas, and unsupervised learning that aims to augment the data efficiency of deep learning is a very promising solution to this challenge. Another challenge is the selection of hyperparameters. Due to the limited computation resources, we have not performed a thorough investigation. Automatic adjustment and some machine learning-inspired algorithms are very promising for addressing this problem, which will be considered in our future work.

We hope that our research will be helpful in addressing the difficulty in developing automation-assisted cervical cancer screening systems; we also believe that this approach can be applied to other medical image processing applications. Future studies on this topic are therefore needed to establish a more robust automation-assisted cervical cancer screening system with satisfactory accuracy under limited computing resources.

## Figures and Tables

**Table 1:** Data distribution of the cells in categories of SIPaKMeD dataset.

Categories	Number of Image Samples
Superficial/Intermediate	813
Parabasal	787
Metaplastic	793
Koilocytotic	825
Dyskeratotic	813
Total	4049

**Table 2:** The classification performances of four lightweight CNNs with traditional loss and hybrid loss.

		Accuracy	Precision	Recall	Specificity
Squeezenet	Traditional loss	93.85	93.96	93.87	98.46
	Hybrid loss	94.52	94.63	94.54	98.62
MobilenetV2	Traditional loss	87.35	87.52	87.41	96.84
	Hybrid loss	83.10	83.41	83.22	95.78
ShufflenetV2	Traditional loss	95.61	95.66	95.61	98.89
	Hybrid loss	96.18	96.30	96.23	99.08
Ghostnet	Traditional loss	95.45	95.52	95.44	98.85
	Hybrid loss	96.39	96.42	96.39	99.09

**Table 3:** Comparison results of the proposed method with existing methods for the SIPaKMeD dataset.

	Accuracy	Total Parameters	Total Memory (M)	Total Flops (GB)
Alexnet [20]	93.58	6.11 × 10^7^	4.19 M	0.70 GB
VGG [39]	95.35	13.84 × 10^7^	109.39 M	15.50 GB
Resnet-101 [31]	94.86	4.45 × 10^7^	161.75 M	7.84 GB
Densenet-121 [32]	96.79	0.80 × 10^7^	147.10 M	2.88 GB
Densenet-121+GCN [25]	98.37	p*	m*	f*
ShufflenetV2+HL	96.18	0.13 × 10^7^	20.84 M	0.15 GB
Ghostnet+HL	96.39	0.40 × 10^7^	40.05 M	0.15 GB

Notes: p* > 0.80 × 10^7^, m* > 147.10 M, f* > 2.88 GB; ShufflenetV2 + HL indicates ShufflenetV2 trained with the proposed hybrid loss function; Ghostnet + HL indicates Ghostnet trained with the proposed hybrid loss function.

## Data Availability

Publicly available datasets were analyzed in this study. This data can be found here: https://www.cs.uoi.gr/~marina/sipakmed.html (accessed on 21 March 2022).
